# Development and validation of a predictive model for cognitive frailty in community-dwelling older adults: a cross-sectional study

**DOI:** 10.3389/fpubh.2025.1673370

**Published:** 2025-12-19

**Authors:** Liwei Sun, Yu Zhang, Wenting Ji, Jia Zheng, Guohua Zheng, Jie Xia

**Affiliations:** 1 School of Nursing and Health Management, Shanghai University of Medicine and Health Sciences, Shanghai, China; 2 School of Nursing, Shanghai University of Traditional Chinese Medicine, Shanghai, China

**Keywords:** cognitive frailty, cognitive frailty screening, community-dwelling older adults, cross-sectional study, prediction model

## Abstract

**Background:**

Cognitive frailty (CF) is a geriatric syndrome involving both physical frailty and cognitive impairment, increasing vulnerability to adverse health outcomes. However, practical prediction models integrating easily accessible, modifiable risk factors for community settings are lacking. This study aimed to develop and validate a predictive model for CF in community-dwelling older adults.

**Methods:**

A cross-sectional study was conducted from September 2022 to May 2024 in Pudong New District, Shanghai, with 979 participants aged 60 and above. Data on sociodemographic characteristics, behavioral factors, nutritional status, sleep quality, depression, as well as lifestyle, health-related factors, physical frailty, and cognitive function were collected through questionnaires administered to older adults, with 20 indicators analyzed. The population was divided into a 7:3 ratio for training and validation. LASSO regression and multivariate logistic regression identified risk factors, and a nomogram prediction model was developed. Model performance was evaluated using ROC curves, calibration curves, and decision curve analysis (DCA).

**Results:**

Of the 979 participants, 31.1% were diagnosed with CF. Seven predictors, including marital status, smoking, Timed Up and Go test (TUGT), depression, sleep quality, nutrition, and medication count, were identified to construct the model. Together, these variables provide a comprehensive assessment of the risk of cognitive frailty in older adults. The model exhibited good predictive performance, with AUC values of 0.753 and 0.733 for the development and validation sets, respectively. The *p*-values for the Hosmer-Lemeshow test were 0.507 and 0.537 for the training and validation cohorts, respectively, indicating a notable calibration curve fit. The DCA curves also show that the model has good predictive ability and stability.

**Conclusion:**

Community-dwelling older adults have a higher incidence of cognitive frailty. This study developed an effective, low-cost, and non-invasive model with promising predictive capabilities that can be used as a screening tool to identify community-dwelling older adults at high risk for cognitive frailty in clinical practice. This model is expected to assist healthcare professionals in improving the effectiveness of prevention of cognitive frailty in community-dwelling older adults.

## Background

With the number of people aged 60 and older growing, the burden of dementia or frailty syndrome in older people are expected to be the most frightening and expensive consequences of increased longevity. As a common geriatric syndrome, cognitive frailty (CF) involves simultaneously the physical frailty and cognitive impairment (1). It renders individuals more vulnerable to adverse health outcomes through generally subtle and progressive physical changes, and has attracted the attention of the medical and scientific communities and public health authorities in many countries (2–4). A recent systematic review of 24 included studies involving 73,643 participants revealed that the pooled prevalence of cognitive frailty in community-dwelling older adults aged 60 years and older was 9% (95%CI: 8% ~ 11%), with an annual trend of increase (5). And the prevalence of CF was higher in older adults with chronic diseases (6, 7). Furthermore, CF is associated with adverse health outcomes including falls, hospitalization, poor quality of life, and mortality (8). However, the development of cognitive frailty is complex, dynamic and reversible (1, 9). Early intervention may delay or even reverse the onset and progression of CF (9). Therefore, development of a predictive model of cognitive frailty is crucial and will provide new opportunities to promote interventions.

Previous studies have reported that various factors, including demographic factors (e.g., age, marital status, education), lifestyle factors (e.g., smoking, physical activity, dietary habits), health conditions and some potential genetic factors, contribute to the onset of cognitive frailty (10). A recent systematic review showed some social factors, such as participation, household, network, and habitat, also influence the progression of CF (11). Modifiable risk factors such as malnutrition, physical inactivity, sedentary behavior, substance use and depression, have also been identified (12). There are a few studies that have attempted to develop risk prediction models for cognitive frailty in older adults with different health conditions (13). A systematic review critically appraised the reported multivariable prediction models in older adults with CF and showed that the usefulness of these models needs to be determined due to methodological limitations and incomplete presentation (14). Existing research on cognitive frailty has identified various risk factors, yet the development of practical prediction models for community use remains limited. Huang et al. (15) developed a predictive model for cognitive frailty in older adults, but it was based on a secondary analysis of a large database and lacked key modifiable factors such as depression and comprehensive sleep assessment. Similarly, Peng et al. (16) constructed a model for older adults patients with multimorbidity, yet the predictors of this model were chosen through univariable analysis, and no external validation was performed, which may result in incorrect predictor selection or variable omission. While these studies provide valuable insights for identifying individuals at risk of cognitive frailty, the reliability and generalizability of the proposed models are constrained by methodological limitations such as insufficient validation and the omission of easily measurable, modifiable predictors. Therefore, there is a clear need for more robust and well-validated prediction models that incorporate readily accessible risk factors to improve early detection and intervention in community settings. This study aims to identify and incorporate the modifiable, accessible and non-invasive factors to construct a risk prediction model appropriate for community-dwelling older adults, that will provide valuable insights for early CF screening and intervention by healthcare professionals in community-dwelling older adults.

## Materials and methods

### Study design and participants

A cross-sectional study was conducted from September 2022 to May 2024 in Pudong New District, Shanghai, China. This study followed the Transparent Reporting of a Multivariable Prediction Model for Individual Prognosis or Diagnosis + AI (TRIPOD + AI) guidelines (17) (Supplementary TRIPOD + AI checklist).

Inclusion criteria: older adults over 60 years old living in the community who voluntarily participated in the study and signed an informed consent. Exclusion criteria: older adults with mental illness (e.g., depression, schizophrenia, dementia, stroke, or epilepsy); those who were unable to cooperate with study procedures (e.g., due to disorders of consciousness, eye disease, deafness, aphasia, etc.); and those with motor dysfunction (i.e., serious infections, malignancies leading to heart, liver, kidney, or other organ failure, and physical dysfunction).

**Figure 1:**
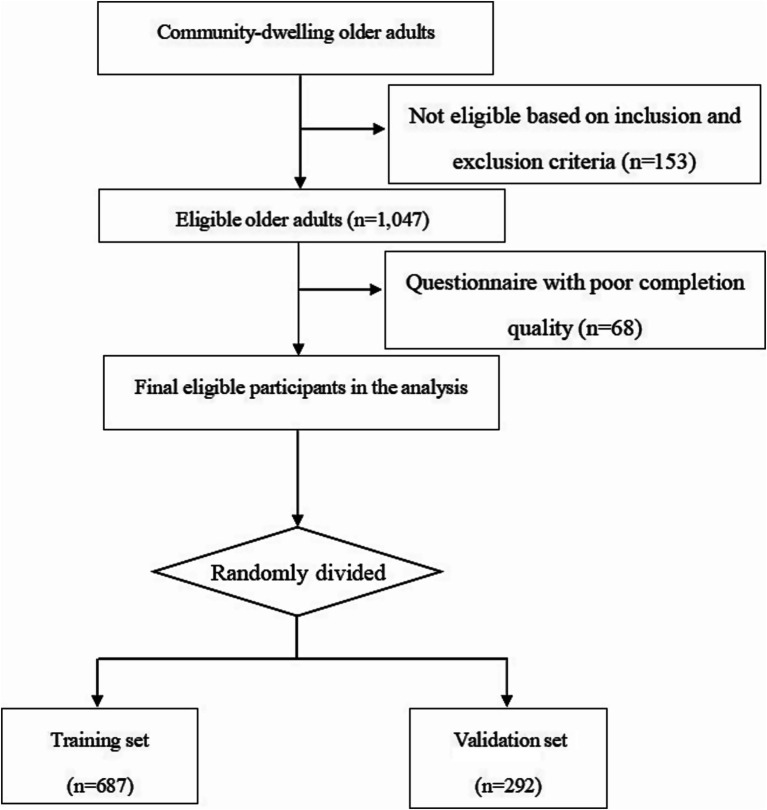
Flow chart of participants into the study.

According to the Tool to Assess Risk of Bias and Application of Prediction Model Studies (PROBAST) (18), a minimum of 20 events per variable (EPVs) was suggested for model development and at least 100 participants with the outcome for model validation to minimize overfitting. More than 400 older adults with CF must be in the development cohort, and there should be at least 100 participants with CF in the validation cohort. A total of 1,200 community-dwelling older adults participated in the screening, and 979 eligible older adults were included in this study (Figure 1).

This study was approved by the ethics committee of Shanghai University of Medicine and Health Sciences (NO. 2022-ZGH-013).

### Assessment of cognitive frailty

Cognitive ability was assessed using the revised Beijing version of the Montreal Cognitive Assessment (MoCA) (19), which consist of 8 cognitive domains: visuospatial/executive function, naming, memory, attention, language, abstraction, delayed recall, and orientation, with a scores range from 0 to 30 and less than 26 scores indicating cognitive impairment. For participants with less than 12 years of schooling, an additional point was added to correct for educational differences. The Cronbach’s *α* of the Chinese version of the MoCA was 0.807 with good reliability and validity (20).

Dementia was assessed using the Clinical Dementia Rating (CDR) scale, which consists of six domains: memory, orientation, judgment and problem solving, community affairs, home and hobbies, and personal care, with a 5-point scale (21). A CDR of 0 indicates no dementia (22).

Physical frailty was evaluated using the modified Edmonton Frailty scale (EFs), which assesses 9 dimensions including cognitive ability, general health status, physical independence, social support, medication use, nutritional status, emotional perception, control, and functional performance, totaling 17 points. Higher scores indicate greater frailty, with a score ≥ 5 scores indicating physical frailty (23). The Cronbach’s alpha coefficient of the Chinese version of the EFS was 0.599 (24). In this study, CF was defined as the coexistence of physical frailty and cognitive impairment to the exclusion of dementia (i.e., MoCA scores < 26, EFs ≥ 5 scores, and CDR < 0.5 scores).

### Candidate predictors

Twenty candidate predictors were selected to screen for the construction of the nomogram in this study, selected based on prior literature and clinical relevance to cognitive frailty (CF) (15, 16). Candidate predictors encompassed multiple domains, including the sociodemographic characteristics, lifestyle and behavioral habits, physical function indicators, psychological variables and other modifiable factors, with the characteristics of being modifiable, readily available, and non-invasive.

### General information questionnaire

The survey questionnaire, was developed through an extensive literature review and collaboration between researchers and experts. The demographic characteristics, lifestyle and behavioral habits of the participants were investigated by the trained researchers using a self-designed questionnaire, which consisted of the sociodemographic characteristics (e.g., age, gender, height, weight, education, marital status, and residence status), lifestyle and behavioral habits (e.g., tobacco and alcohol use, physical activities, leisure activities, puzzle activities); and health condition (suffering from chronic diseases, medication). The full wording of all questionnaire items, response options is provided in Supplementary Table S1: General Information Questionnaire.

### Physical performance assessment

Physical performance was assessed using the Timed Up and Go test (TUGT). In the TUG test, the participant was asked to stand up from a sitting position on a chair with approximately 45 cm high, walk 3 meters at a normal pace in a straight line to a mark, turn around, walk back to the chair and then sit down on it. The time the participant took to complete this test was timed to 1 s with a stopwatch (25). Two measurements were taken at 30-s intervals, and the mean of these measurements was used for statistical analysis. Shorter times indicate better physical performance in gait and balance function. Participants with a final test time of less than 10 s are considered to be able to move freely; 10–19 s indicates mostly independent movement; 20–29 s indicates impaired movement (26).

### Depression assessment

The Geriatric Depression Scale (GDS-15), which is widely recognized for its validity and appropriateness for older adults, was used to assess participant’s depression (27, 28). This scale consists of 15 items with responses categorized as “yes/no.” Each affirmative response scores 1 point, resulting in a total score ranging from 0 to 15. Higher scores indicate more severe depressive symptoms, with a total score of 8 or higher indicating the presence of depressive symptoms. The scale demonstrated acceptable internal consistency (Cronbach’s *α* = 0.745) and good discriminant validity for measuring depressive symptoms in Chinese older adults (29).

### Sleep quality assessment

The Pittsburgh Sleep Quality Index (PSQI) questionnaire is used to assess participants’ sleep quality (30). The PSQI consists of 18 items in seven domains: subjective sleep quality, sleep latency, sleep duration, sleep efficiency, sleep disturbances, use of sleep medications, and daytime dysfunction. Each item is rated on a scale of 0 to 3, contributing to a total PSQI score that ranges from 0 to 21. Scores below 7 indicate very good sleep quality, while scores of 7 or higher suggest the presence of sleep disorders. The internal consistency of the Chinese version of the PSQI was found to be acceptable (Cronbach’s *α* = 0.68) in a previous study involving community-dwelling centenarians (31).

### Nutritional assessment

The Mini Nutrition Assessment Short-Form (MNA-SF) (32) was used for the assessment of participants’ nutritional status on the basis of six primary factors: body mass index, recent weight loss, acute illness or stress, mobility status, cognitive status, and appetite problems. Scores on the MNA-SF range from 0 to 14, with score of 12–14 indicating good nutritional level and scores ≤11 indicating malnutrition or risk of malnutrition (33). The Mini-Nutritional Assessment Short-Form (MNA-SF) demonstrated acceptable internal consistency, with a Cronbach’s alpha of 0.77 (34).

### Sample size

According to the Tool to Assess Risk of Bias and Application of Prediction Model Studies (PROBAST) (18), a minimum of 20 events per variable (EPVs) was suggested for model development and at least 100 participants with the outcome for model validation to minimize overfitting. More than 400 older adults with CF must be in the development cohort, and there should be at least 100 participants with CF in the validation cohort.

### Missing data

Missing data arose from item non-response, refusal to answer specific questions, incomplete recall, and occasional non-completion of performance testing due to safety or fatigue. The missing data were dealt with multivariate imputation by chained equation (MICE) if the missing data in each variable were random and accounted for less than 50% (35). Five imputations were generated using multiple chains. The dataset with the lowest Akaike Information Criterion (AIC) value was chosen.

### Statistical methods

Data analysis utilized SPSS 25.0 and R software (version 4.2.2). Categorical variables were expressed as percentages, and group comparisons were made using the *χ*^2^ test or Fisher’s exact test.

The data were randomly divided into training (*n* = 685) and validation (*n* = 294) sets at a 7:3 ratio. Predictor variables included in the column line graph (nomogram) were selected in two steps. First, the training set data were analyzed using LASSO regression to select predictors of cognitive frailty. LASSO analysis is performed by generating a penalty function that is a compression of the coefficients of the variables in the regression model to prevent overfitting and solve the problem of strong covariance. Second, the most important features selected by the LASSO regression from the training set were used in a multifactor logistic regression analysis. Variables with *p* < 0.05 were included in the nomogram, and the multifactorial analysis was used to predict the respective probabilities of an individual experiencing cognitive frailty. Performance of the nomogram was evaluated using the receiver operating characteristic (ROC) curve and calibration curve. The area under the ROC curve (AUC) ranged from 0.5 (no discrimination) to 1 (perfect discrimination). Additionally, decision curve analysis (DCA) was conducted to establish the net benefit prediction threshold.

## Results

### Baseline characteristics

A total of 979 community-dwelling older adults aged 60 years and above were included in this study. Among these participants, 42.19% (*n* = 413) were male and 57.81% (*n* = 566) were female, 31.05% (*n* = 304) were categorized as cognitive frailty (CF) and 68.95% (*n* = 675) were non-cognitive frailty (non-CF). The dataset was divided into a training set comprising 687 cases and a validation set with 292 cases.

Significant differences were observed between the CF and non-CF groups in terms of age, marital status, current smoking, use of electronic devices, TUGT (Timed Up and Go Test), depression, sleep quality, nutritional level, number of chronic diseases, and number of medications (*p* < 0.05) (Table 1).

**Table 1:** Baseline characteristics of the study population.

Variables	Total (*n* = 979)	Non-CF (*n* = 675)	CF (*n* = 304)	*χ*^2^ value	*p* value
Gender, *n* (%)				0.10	0.754
Male	413 (42.19)	287 (42.52)	126 (41.45)		
Female	566 (57.81)	388 (57.48)	178 (58.55)		
Age, *n* (%)				17.97	<0.001
60–70	625 (63.84)	458 (67.85)	167 (54.93)		
70–80	279 (28.50)	177 (26.22)	102 (33.55)		
>80	75 (7.66)	40 (5.93)	35 (11.51)		
BMI, *n* (%)				4.08	0.253
≤18.5	37 (3.78)	28 (4.15)	9 (2.96)		
18.5–24	579 (59.14)	403 (59.70)	176 (57.89)		
24–28	311 (31.77)	204 (30.22)	107 (35.20)		
≥28	52 (5.31)	40 (5.93)	12 (3.95)		
Marital status, *n* (%)				24.12	<0.001
Married/Remarried	863 (88.15)	618 (91.56)	245 (80.59)		
Unmarried/Widowed/Divorced	116 (11.85)	57 (8.44)	59 (19.41)		
Residence status, *n* (%)				0.22	0.637
Solitude	110 (11.24)	78 (11.56)	32 (10.53)		
Cohabitation	869 (88.76)	597 (88.44)	272 (89.47)		
Economic income, *n* (%)				4.87	0.088
≤ 2000 (Yuan/Month)	205 (20.94)	131 (19.41)	74 (24.34)		
2000–6,000 (Yuan/Month)	696 (71.09)	484 (71.70)	212 (69.74)		
≥ 6,000 (Yuan/Month)	78 (7.97)	60 (8.89)	18 (5.92)		
Current smoking, *n* (%)				4.61	0.032
No	802 (81.92)	541 (80.15)	261 (85.86)		
Yes	177 (18.08)	134 (19.85)	43 (14.14)		
Current drinking, *n* (%)				2.20	0.138
No	825 (84.27)	561 (83.11)	264 (86.84)		
Yes	154 (15.73)	114 (16.89)	40 (13.16)		
Watching TV listening to the radio, *n* (%)				1.28	0.258
Never participates	109 (11.13)	70 (10.37)	39 (12.83)		
≤2/2–3/>3(h/day)	870 (88.87)	605 (89.63)	265 (87.17)		
Puzzle activities, *n* (%)				2.28	0.131
Never participates	464 (47.40)	309 (45.78)	155 (50.99)		
≤2/2–3/>3(h/day)	515 (52.60)	366 (54.22)	149 (49.01)		
Use of electronics status, *n* (%)				8.57	0.003
Never participates	220 (22.47)	134 (19.85)	86 (28.29)		
≤2/2–3/>3(h/day)	759 (77.53)	541 (80.15)	218 (71.71)		
Other leisure activities, *n* (%)				3.29	0.070
Never participates	720 (73.54)	508 (75.26)	212 (69.74)		
≤2/2–3/>3(h/day)	259 (26.46)	167 (24.74)	92 (30.26)		
TUGT, *n* (%)				71.59	<0.001
0–10	464 (47.40)	368 (54.52)	96 (31.58)		
10–20	464 (47.40)	293 (43.41)	171 (56.25)		
≥20	51 (5.21)	14 (2.07)	37 (12.17)		
Years of education, *n* (%)				0.87	0.647
≤6	337 (34.42)	226 (33.48)	111 (36.51)		
6–12	511 (52.20)	358 (53.04)	153 (50.33)		
≥12	131 (13.38)	91 (13.48)	40 (13.16)		
Physical activity level, *n* (%)				2.70	0.259
Low level	254 (25.94)	178 (26.37)	76 (25.00)		
Moderate level	542 (55.36)	363 (53.78)	179 (58.88)		
High level	183 (18.69)	134 (19.85)	49 (16.12)		
Depression, *n* (%)				22.84	<0.001
No	790 (80.69)	572 (84.74)	218 (71.71)		
Yes	189 (19.31)	103 (15.26)	86 (28.29)		
Sleep quality, *n* (%)				30.06	<0.001
Well	832 (84.98)	602 (89.19)	230 (75.66)		
Bad	147 (15.02)	73 (10.81)	74 (24.34)		
Nutritional level, *n* (%)				38.48	<0.001
Well	692 (70.68)	518 (76.74)	174 (57.24)		
Bad	287 (29.32)	157 (23.26)	130 (42.76)		
Number of chronic diseases, *n* (%)				11.11	0.004
0	163 (16.65)	125 (18.52)	38 (12.50)		
1–2	778 (79.47)	531 (78.67)	247 (81.25)		
≥3	38 (3.88)	19 (2.81)	19 (6.25)		
Number of medications, *n* (%)				57.24	<0.001
0	356 (36.36)	293 (43.41)	63 (20.72)		
1–3	542 (55.36)	345 (51.11)	197 (64.80)		
≥4	81 (8.27)	37 (5.48)	44 (14.47)		

### Predictive model development

**Figure 2:**
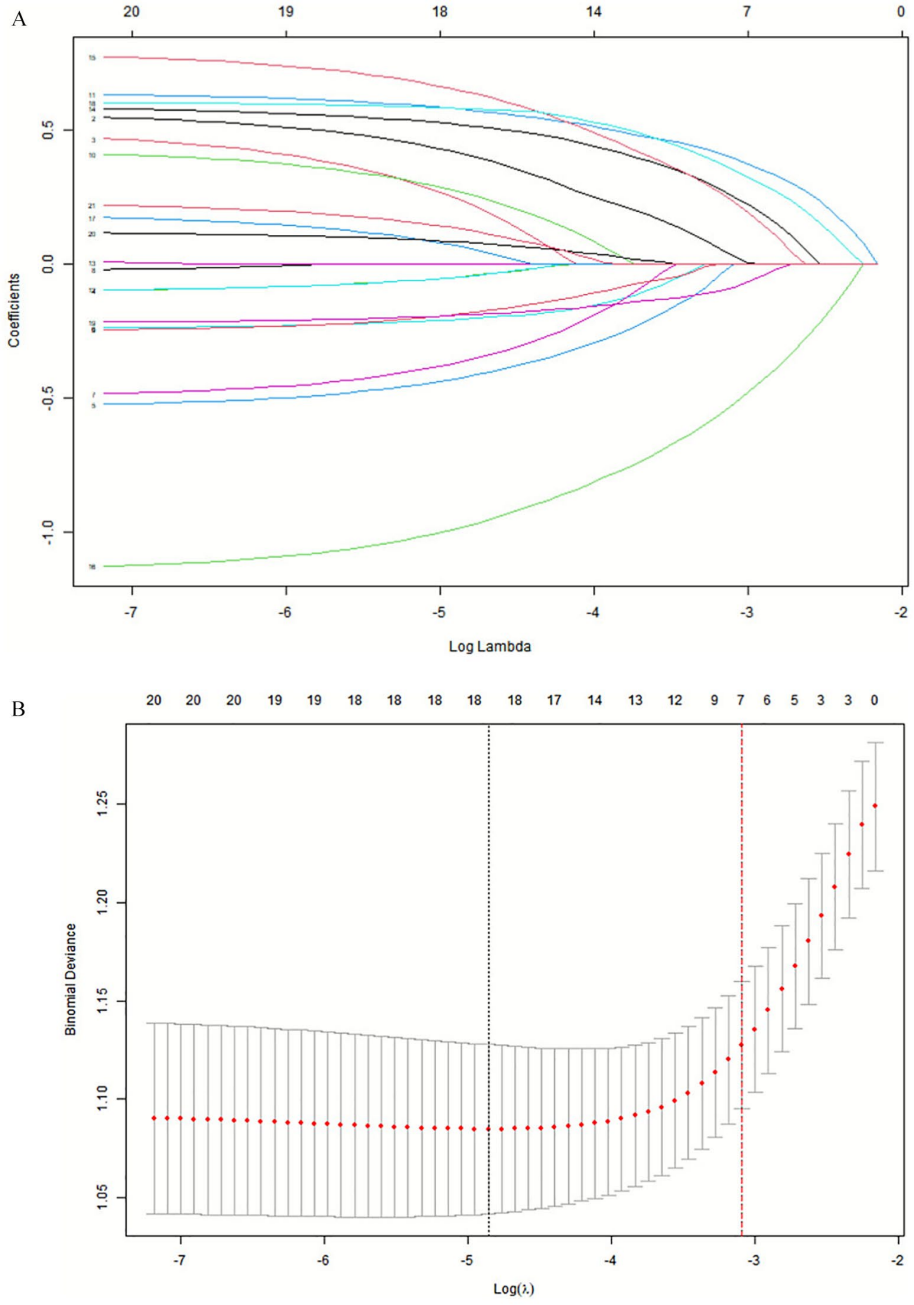
Demographic characteristics and variables related CF selection using the LASSO regression model. **(A)** A coefficient profile was generated according to the logarithmic (lambda) sequence, and non-zero coefficients were produced by the optimal lambda. **(B)** The optimal parameter (lambda) in the LASSO model was selected by tenfold cross-validation using minimum criteria. The partial likelihood deviation (binomial deviation) curve relative to log (lambda) was plotted. A virtual vertical line was drawn at the optimal value using one SE of minimum criterion (the 1-SE criterion).

Based on the variables in the training set, Lasso regression analysis and ten-fold cross-validation were performed on 20 predictor variables, and the results determined seven non-zero characteristic variables at one standard error from the minimum model mean square error (*λ* = 0.038), including marital status, current smoking, TUGT, depression, sleep quality, nutritional level, and number of medications (Figure 2). A logistic regression analysis was conducted with these seven risk factors as independent variables, with CF as the dependent variable, and the results indicated that marital status, TUGT, depression, current smoking, nutritional level, sleep quality, and number of daily medications were independent influencing factors for cognitive frailty (*p* < 0.05) (Table 2). The final prediction model expression is: CF = −1.668 + 0.471 × Marital Status−0.864 × Smoking+0.645 × TUGT+0.700 × Depression+0.732 × Sleep Quality−1.072 × Nutritional Status+0.719 × Number of Medications.

**Table 2:** The prediction model with multivariate logistic regression.

Variables	B	S. E.	*Z*	*p*	OR	95% CI	
						Lower	Upper
Constant	−1.668	0.301	−5.541	<0.001	0.189	0.105	0.340
Marital status	0.471	0.183	2.579	0.010	1.602	1.120	2.291
Current smoking	−0.864	0.264	−3.271	0.001	0.422	0.251	0.707
TUGT	0.645	0.158	4.080	<0.001	1.906	1.398	2.598
depression	0.700	0.228	3.068	0.002	2.014	1.288	3.149
Sleep quality	0.732	0.252	2.910	0.004	2.080	1.270	3.406
Nutritional level	−1.072	0.199	−5.395	<0.001	0.342	0.232	0.505
Number of medications	0.719	0.158	4.539	<0.001	2.053	1.505	2.800

### Visualization of the predictive model

**Figure 3:**
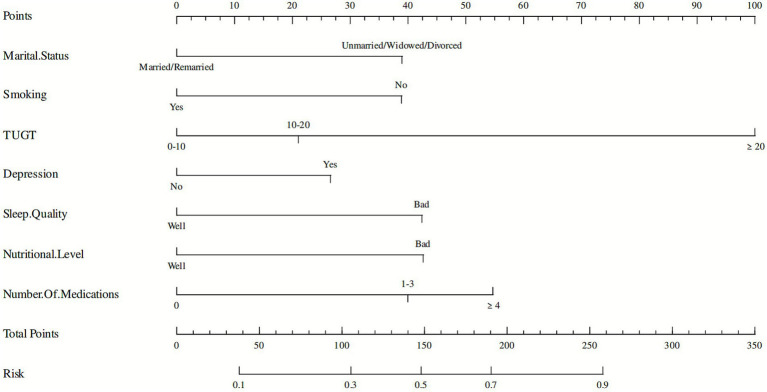
A nomogram for predicting cognitive frailty in the older adults in China.

Based on the factors identified by Lasso regression and multivariate logistic regression analysis, a nomogram for CF was constructed using the “rms” package (Figure 3). This model can be used to predict the risk of CF in community-dwelling older adults individuals aged 60 years and above. In clinical use, based on the general data of the older adults, individual scores corresponding to each independent influencing factor can be obtained using the ruler and then summed to obtain the total score. Finally, the predicted probability of cognitive frailty risk can be obtained by projecting the total score downward on the score axis.

### Predictive model validation

#### Discrimination

**Figure 4:**
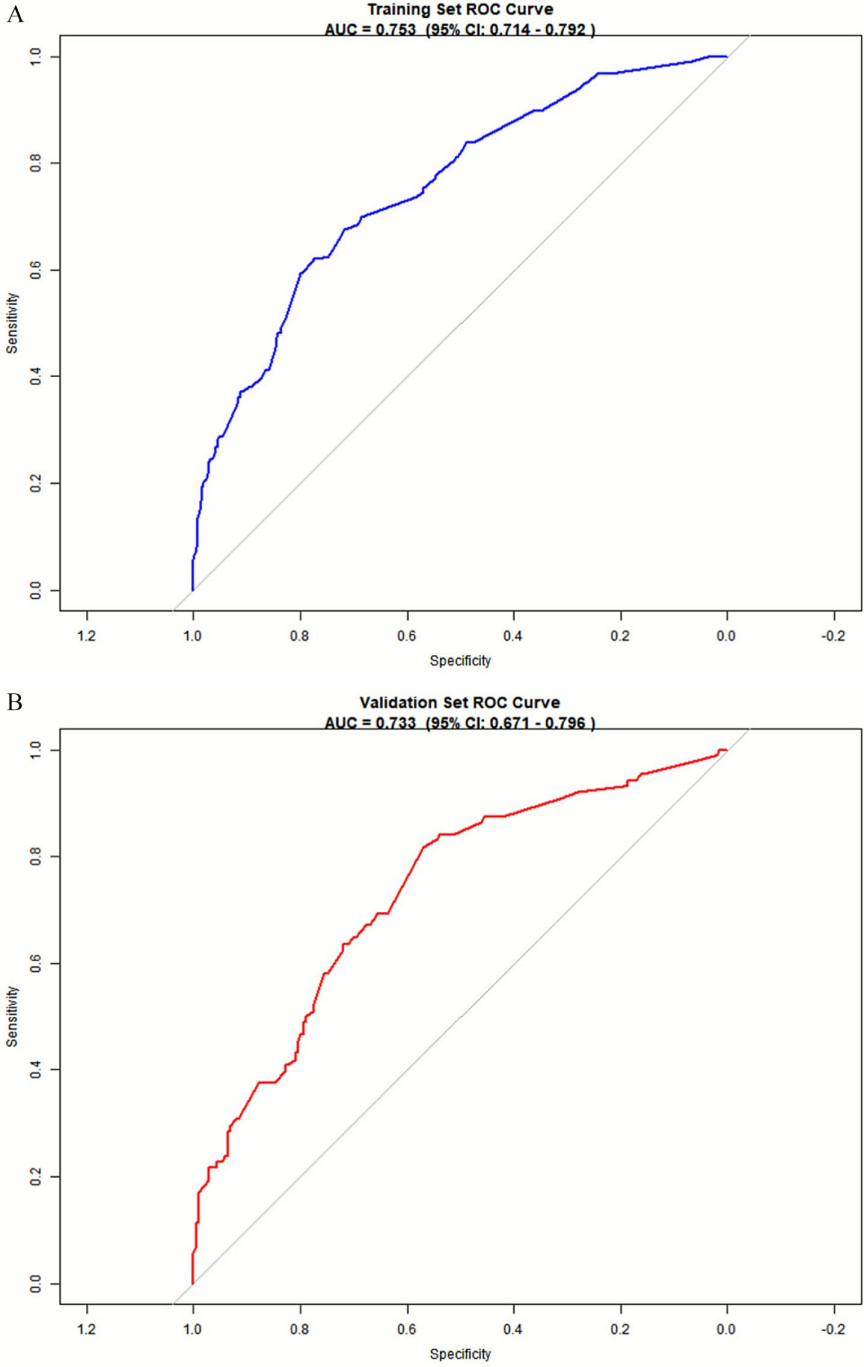
ROC curve of the predictive model. **(A)** Nomogram ROC curves generated from the training dataset. **(B)** Nomogram ROC curves generated using the validation dataset.

The predictive model was applied to the validation set to perform internal validation. ROC curve analysis results showed good predictive power for the nomogram. The results showed that the area under the ROC curve (AUC) for the training set was 0.753 (95% CI = 0.714–0.792), with a cutoff value of 0.354, sensitivity of 62.0% and specificity of 77.5% (Figure 4A). For the validation set, the AUC was 0.733 (95% CI = 0.671–0.796), with a sensitivity of 81.8% and specificity of 56.9% (Figure 4B), indicating that the model can effectively differentiate between CF and non-CF older adults individuals in the community.

#### Calibration of the predictive model

**Figure 5:**
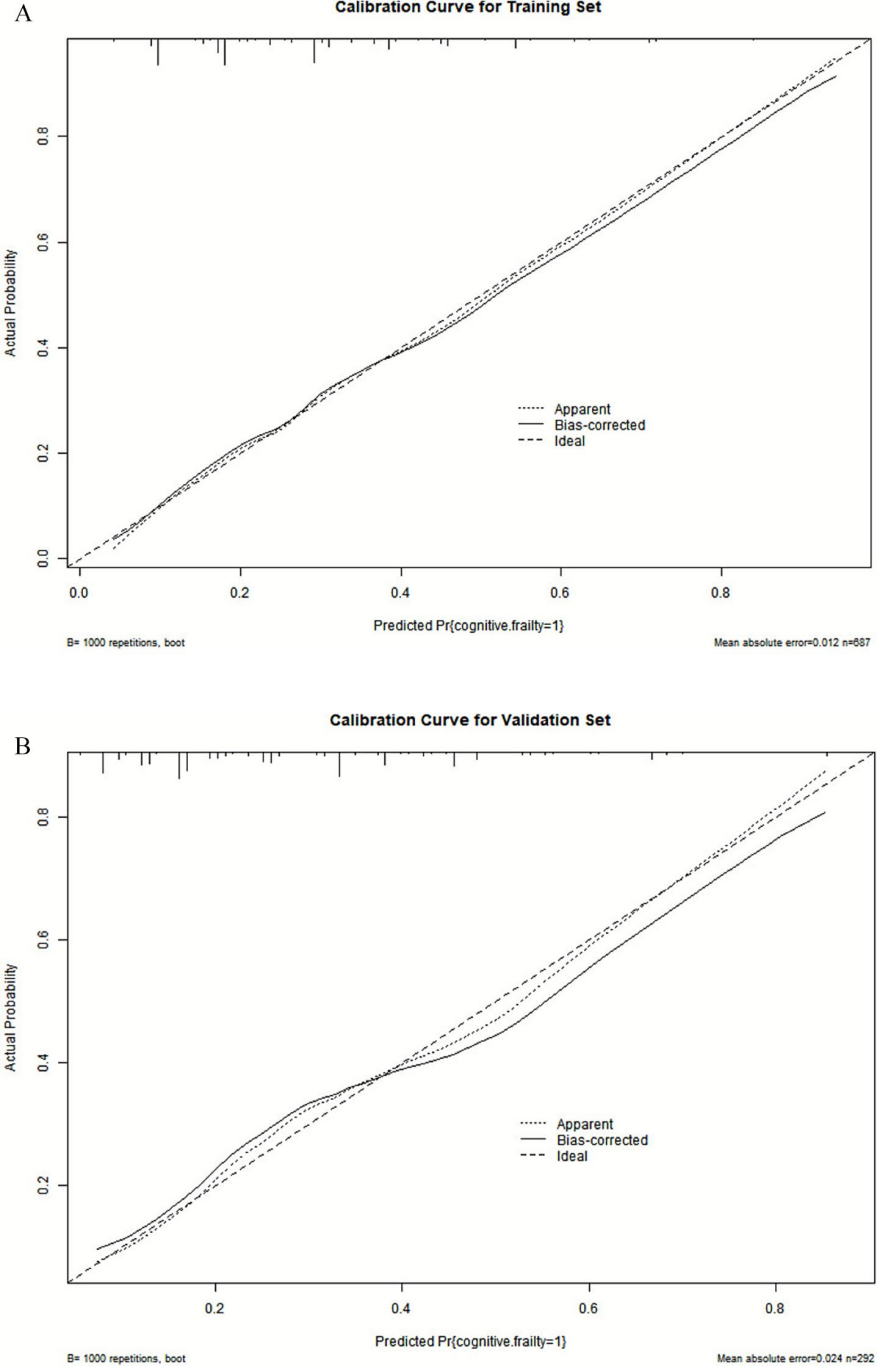
Calibration curve for the nomogram. **(A)** Calibration plots for training dataset. **(B)** Calibration plots for validation dataset.

The Hosmer-Lemeshow (H-L) goodness-of-fit test showed a good model fit with *χ*^2^ = 7.28, *p* = 0.507 for the training set and *χ*^2^ = 6.99, *p* = 0.537 for the validation set. Moreover, the calibration curves of the training and validation sets showed that the predicted and actual curves were generally consistent, indicating good calibration of the model (Figures 5A,B).

#### Evaluation of clinical validity

**Figure 6:**
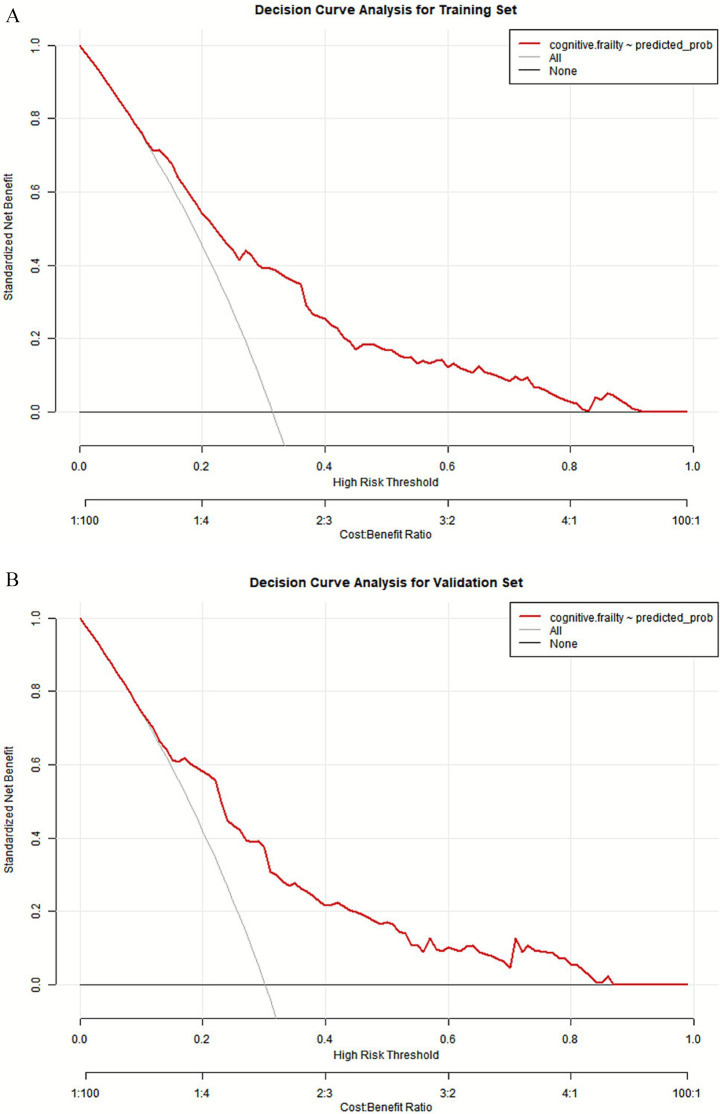
DCA of the nomogram. **(A)** DCA curves for training data set. **(B)** DCA curves for validation data set.

The threshold probability for CF in the training set ranged from 0.00 to 0.91, indicating a wide range of threshold probabilities. The net benefit of using the nomogram risk prediction model to predict cognitive frailty in community-dwelling older adults was relatively high, and this was confirmed in the validation group (Figures 6A,B).

## Discussion

This study developed and validated a predictive model of cognitive frailty (CF) in community-dwelling older adults. The prevalence of CF in this population was found to be 31.1%, which is consistent with previous studies (13). This model identified several risk factors associated with CF in older patients, including marital status, daily medications used, TUGT performance, depression, current smoking, poor sleep quality, and malnutrition, which are easy to obtain and assess, facilitating translation to the community setting. The model also showed good discriminative ability and calibration in both the training and validation datasets, confirming its reliability.

This study identified marital status as a predictor of CF among older adults living in the community. The current single status of older adults in this study was found to be at high risk for developing cognitive frailty compared to their married counterparts, consistent with previous research (36). Older adults with a spouse benefit from increased emotional support, which helps to maintain their vitality levels. In contrast, the single older adults may experience loneliness and depression due to reduced daily communication and social engagement, which affects both physical and cognitive function (37, 38).

Many nutritional components, such as vitamins, minerals and high-quality protein, have been identified for their beneficial effects on cognitive or physical health (39). Nutritional deficiencies can significantly contribute to physical frailty and cognitive decline in older adults (10). Insufficient protein and calorie intake among older adults can result in increased muscle wasting, decreased muscle mass, and heightened risks of falls, disability, and cognitive decline (40, 41). Malnutrition is therefore an important contributing factor in the development of cognitive frailty (42). The current study found a strong correlation between nutritional intake and cognitive frailty, which is consistent with the results of previous studies.

This study shows that depressive symptom in older adults is an independent risk factor of CF, and the community-dwelling older adults with depressive symptoms had 2.014-fold higher odds of cognitive frailty compared to the older adults without depressive symptoms. A previous study found that the risk of cognitive frailty in older adults with depression was 2.01 times higher than in older adults without depression (43), which is consistent with the findings of this study. A recent systematic review also reveals the association between depression and cognitive frailty, suggesting that the two affect each other (44). The possible reason is that they share similar mechanisms of pathophysiology, including inflammation and dysregulation of the hypothalamic–pituitary–adrenal axis (45).

Our study revealed that the physical performance on gait and balance ability measured by the TUG test as a predictor of CF, with 1 s increase in the TUG test significantly increased the risk of developing cognitive frailty 1.906 times. Previous studies have shown that the frail older adults experienced impaired gait and balance function, and those with impaired gait and balance were vulnerable to frailty, so gait and balance assessments can be considered early indicators of physical frailty in older adults (46, 47). In addition, the TUG test has served as a straightforward marker of gait impairment associated with cognitive impairment and dementia, and has been used as a risk indicator for cognitive impairment and dementia in community-dwelling older adults (48, 49).

Sleep quality is considered an independent contributor to CF in this study. Inappropriate sleep duration is a risk factor for developing chronic diseases, and sleep disorder associated with poor health outcomes in older adults (50). Studies have shown that sleep disorder or poor sleep quality can lead to the decreased appetite, inadequate nutritional intake, anxiety, depression, and other mental disorders, ultimately contributing to sarcopenia and cognitive decline in older people (51, 52). Hyperactivation of the hypothalamic–pituitary–adrenal axis, together with testosterone, insulin-like growth factor 1 (IGF-1), and growth hormone, as endocrine mechanisms, may play an intermediate role in the association between sleep quality and frailty (53). In addition, sleep disorders can impair cognitive function through inflammatory responses, vascular changes, dysregulation of beta-amyloid protein, and increased tau protein levels (54).

In addition, this study shows that smoking is associated with CF in older adults. A previous study also reported the association between smoking and increased incidence of CF in older adults (55). Another study found that older smokers had an increased susceptibility to CF, with the risk increasing alongside smoking duration (56). This is attributed to the neurotoxic effects of tobacco products, which increase carbon monoxide levels and cause persistent cerebral hypoxia, greatly increasing the risk of cognitive impairment or physical frailty (57). This study found that the number of daily medications taken was an independent predictor for risk of CF in community-dwelling older adults. It is reasonable that more medications taken would be associated with more chronic diseases and poorer health conditions in older adults (58). The adverse effect of drugs can lead directly to organ damage and various metabolic disorders, including the abnormalities of lipid metabolism and calcium-phosphorus metabolism disorders, which can lead to cognitive impairment and physical frailty in the older adults (59).

The progression of CF in the older adults leads to functional impairment, hospital readmissions, dementia, and mortality, impacting quality of life and placing burdens on caregivers and the healthcare system. Consequently, it assumes paramount importance to construct a predictive model for assessing CF risk and implementing timely interventions. Nevertheless, the factors influencing the occurrence of CF are complex and numerous. Many predictors in previous models rely heavily on specific instruments for detection, making them challenging to implement in community setting. Additionally, some predictors in these models are often difficult to modify, limiting their utility in guiding preventive interventions. Furthermore, existing models, such as those based on the China Health and Retirement Longitudinal Study (CHARLS) database, often fail to account for critical modifiable risk factors such as depression, nutrition, and comprehensive sleep assessments, which are essential for targeted and actionable interventions (15). By including these actionable variables, our model enhances clinical relevance and supports targeted prevention strategies. It is specifically tailored for community-dwelling older adults and uses readily available variables, making it practical for real-world implementation in community health programs.

In this study, we identified smoking, sleep quality, nutrition, depression, gait and balance as the main factors predicting CF in community-dwelling older adults. These factors are non-invasive, low-cost, and easy to obtain, making the model particularly suitable for application in community settings. The prediction model was constructed based on these seven factors associated with the development of CF and showed good discrimination, calibration and clinical validity. By leveraging this model, community health workers or volunteers can efficiently screen large populations using simple assessments without the need for specialized equipment or extensive training. This allows for timely identification of at-risk individuals and facilitates early intervention strategies tailored to modifiable risk factors.

### Limitations

Despite the satisfactory value of our predictive model, it is important to acknowledge certain limitations. Firstly, this was a cross-sectional study, and because exposure and outcomes were assessed simultaneously, it is not possible to determine causal relationships between the factors studied and cognitive frailty. As such, the findings should be interpreted as associations, and longitudinal studies are needed to validate these associations and establish temporal relationships. Secondly, because this study only included participants from the city of Shanghai, China, there may be selection bias, which could limit the applicability of the findings to other settings or populations; therefore, its nationwide or international application needs further external validation by a multicentre study. Thirdly, most of the predictors were self-reported by older adults, which may potentially introduce information bias. Hence, it is warranted that objective predictors assessments be incorporated into future research in order to substantially enhance the study. Furthermore, future studies could employ more advanced machine learning techniques, to further enhance predictive performance and explore the stability of predictor importance in different modeling frameworks.

## Conclusion

This study developed and validated a nomogram model incorporating sleep quality, depression, TUGT performance, smoking, nutritional status, marital status, and medication use to predict the risk of cognitive frailty in community-dwelling older adults. The model demonstrated strong performance in terms of discrimination, calibration, and clinical utility, proving its reliability and real-world applicability.

The nomogram model developed in this study serves as a foundation for enhancing health management strategies for older adults at risk of cognitive frailty. It provides scientific support for the development of effective prevention and intervention measures, ensuring early identification and targeted care for high-risk individuals.

## Data Availability

The raw data supporting the conclusions of this article will be made available by the authors, without undue reservation.
