# Supplementary material for: Development and validation of a predictive model for cognitive frailty in community-dwelling older adults: a cross-sectional study
Source: Front Public Health. 2025 Dec 19;13:1673370. doi: 10.3389/fpubh.2025.1673370 (PMC12757231; doi:10.3389/fpubh.2025.1673370)
Supplement: Supplementary file 2 [file Supplementary_file_1.docx]

**General Information Questionnaire**

1. Name: ____________ Gender: ☐ Male ☐ Female Age: ______ (years)

2. Current place of residence: __________________________ Length of residence: ______ (years)

3. Contact information: ________________________________

4. Height (cm): ______ Weight (kg): ______

5. Marital status: ☐ Married ☐ Widowed ☐ Divorced ☐ Remarried ☐ Unmarried

6. Residence status (living arrangement): ☐ Living alone ☐ Cohabitation

7. Monthly economic income (RMB): ☐ <2000 ☐ 2000–6000 ☐ >6000

8. Years of education: ☐ ≤6 ☐ 6–12 ☐ ≥12

9. Smoking and alcohol use:
 • Smoking: ☐ Yes, ____ cigarettes/day ☐ No
 • Alcohol: ☐ Yes, ____ times/day ☐ No
 (a) Per WHO (1984): a smoker is defined as ≥1 cigarette/day for ≥1 year (cumulative or continuous).
 (b) Per the 2016 Chinese Dietary Guidelines: adult men ≥25 g/day alcohol, adult women ≥15 g/day, qualify as drinkers.

10. Current health status:
 (1) Number of medications currently taken: ______
 (2) Current chronic diseases (please specify): _________________________________

11. Use of electronic devices (hours/day): ☐ Never ☐ ≤2 ☐ 2–3 ☐ ≥3

12. Puzzle activities (hours/day): ☐ Never ☐ ≤2 ☐ 2–3 ☐ ≥3

13. Watching TV / Listening to the radio (hours/day): ☐ Never ☐ ≤2 ☐ 2–3 ☐ ≥3

14. Other leisure activities (hours/day): ☐ Never ☐ ≤2 ☐ 2–3 ☐ ≥3
